# Cellular Analysis and Chemotherapeutic Potential of a Bi-Functionalized Halloysite Nanotube

**DOI:** 10.3390/pharmaceutics12100962

**Published:** 2020-10-13

**Authors:** Yangyang Luo, Ahmed Humayun, Teresa A. Murray, Benjamin S. Kemp, Antwine McFarland, Xuan Liu, David K. Mills

**Affiliations:** 1 Molecular Sciences & Nanotechnology, Louisiana Tech University, Ruston, LA 71272, USA; yangyang317luo@gmail.com (Y.L.); ah.humayun@gmail.com (A.H.); tmurray@latech.edu (T.A.M.); bscott.kemp@gmail.com (B.S.K.); awm011@latech.edu (A.M.); Xliu@latech.edu (X.L.); 2 School of Biological Sciences and the Center for Biomedical Engineering, Louisiana Tech University, Ruston, LA 71272, USA

**Keywords:** targeted drug delivery, halloysite nanotube, osteosarcoma, methotrexate, surface modification

## Abstract

The surface of halloysite nanotubes (HNTs) was bifunctionalized with two ligands—folic acid and a fluorochrome. In tandem, this combination should selectively target cancer cells and provide a means for imaging the nanoparticle. Modified bi-functionalized HNTs (bi-HNTs) were then doped with the anti-cancer drug methotrexate. bi-HNTs were characterized and subjected to in vitro tests to assess cellular growth and changes in cellular behavior in three cell lines—colon cancer, osteosarcoma, and a pre-osteoblast cell line (MC3T3-E1). Cell viability, proliferation, and cell uptake efficiency were assessed. The bi-HNTs showed cytocompatibility at a wide range of concentrations. Compared with regular-sized HNTs, reduced HNTs (~6 microns) were taken up by cells in more significant amounts, but increased cytotoxicity lead to apoptosis. Multi-photon images confirmed the intracellular location of bi-HNTs, and the method of cell entry was mainly through caveolae-mediated endocytosis. The bi-HNTs showed a high drug loading efficiency with methotrexate and a prolonged period of release. Most importantly, bi-HNTs were designed as a drug carrier to target cancer cells specifically, and imaging data shows that non-cancerous cells were unaffected after exposure to MTX-doped bi-HNTs. All data provide support for our nanoparticle design as a mechanism to selectively target cancer cells and significantly reduce the side-effects caused by off-targeting of anti-cancer drugs.

## 1. Introduction

Cancer is the second leading cause of death in the United States [1]. While radiation and surgery treatments have advanced cancer treatment, chemotherapy is still one of the leading treatment modalities [2]. Unfortunately, current chemotherapeutic agents adversely affect healthy cells at the target site and elsewhere in the body [3]. Chemotherapy drugs work by impairing cell division and are effective treatments for early-stage tumors when cancer cells are rapidly multiplying. However, they also produce a range of unpleasant side effects. Systemic toxicity is an undesired consequence for most chemotherapeutic drugs [1,2]. The development of a multi-functional drug delivery system (DDS) with an ability to provide extended, controlled, and selective drug release is at the forefront of current cancer therapy research [2,3]. Targeting chemotherapeutic drugs directly at the tumor cells would increase drug effectiveness and reduce side effects.

Methotrexate (MTX) is a folic acid antagonist. It has an anti-cancer effect on lymphoblastic leukemia, lymphoma, osteosarcoma, and breast, lung, head, and neck cancers [4,5,6]. MTX restricts cancer cell growth by disrupting transmethylation reactions, which are essential in forming proteins, lipids, and myelin [7]. However, the high dose administration of MTX can damage cells in bone marrow, gastrointestinal mucosa, and hair follicles. Severe side effects, including renal failure, neurotoxicity, hematologic toxicity, mucocutaneous toxicity, and pulmonary toxicity, may result after high dose MTX treatments [8]. Accordingly, a targeted MTX drug delivery system designed to improve its target delivery and reduce destruction to healthy cells seems like a promising approach.

Halloysite nanotubes (HNTs) have attracted significant attention in drug delivery due to its biocompatibility, physicochemical stability, and unique structural properties. HNTs are naturally occurring nanoscale tubes composed of Al_2_O_3_·2SiO_2_·*n*H_2_O. In the process of aluminosilicate rolling, Al–OH groups are folded inside, and Si-O-Si groups are exposed to the outer surface. At neutral pH, the inner surface of HNTs is positively charged, and the outer surface is negatively charged [9]. Depending on the geological origin, the lumen and outer diameters of HNTs range between 10–15 nm and 50–80 nm, respectively [9]. While the length of HNTs averages between 0.5–2 μm. With specific surface modification, active agents can be conjugated on the surface or encapsulated in the empty HNT lumen. Curcumin [10], doxorubicin [11], irinotecan [12], and resveratrol [13] have been successfully doped into HNTs and shown to have an anti-cancer effect.

In a previous study, we demonstrated that the HNT surface could be functionalized with *N*-[3-(trimethoxysilyl)propyl) ethylenediamine (DAS) for grafting folic acid (FA) and fluorescein isothiocyanate (FITC) resulting in a bifunctionalized HNTs (bi-HNT). We used FA as a ligand for targeted drug delivery and FITC as a visual tracking agent. Surface modification using FA and FITC was further confirmed by FTIR, 13C CPMAS NMR spectrum, and UV-Vis. Folic acid directed HNTs adhered to folate receptors, which are overexpressed in numerous cancers but rarely expressed or nonexistent in most normal tissues [14,15,16].

In this study, we focused on identifying the cellular uptake mechanism and intracellular location of bi-HNTs after endocytosis. We further studied cellular interactions after exposure to bi-HNTs, including cell viability, proliferation, and cell uptake efficiency. When colon cancer cells (CT26WT) were co-cultured with bi-HNTs, cell viability decreased at higher doses. Further analysis showed that cell death was due to apoptosis. Also, size reduced bi-HNTs resulted in higher cell mortality. MTX loaded into bi-HNTs (MTX-bi-HNTs) was analyzed for its specific targeting ability in vitro after co-culturing with three different cell types. Our results show that bi-HNTs provided a high loading capacity for the cytotoxic cancer drug MTX and, bi-HNTs specifically targeted osteosarcoma cells and released MTX and inhibited cell proliferation. Interestingly, in the presence of MTX, non-osteosarcoma cells were unaffected.

## 2. Materials and Methods

### 2.1. Materials

All cell culture materials and reagents (including MTX, chlorpromazine and filipin) were purchased from Sigma Aldrich, St. Louis, MO, USA. Cell culture dishes, pipettes and other disposable plastics were purchased from Mid Sci, St. Louis, MO, USA. The *MTT* and XTT Cell Viability Assay Kit was obtained from Biotium, New York, NY, USA. The Annexin V-FITC Apoptosis Kit was purchased from Enzo Life Sciences, Inc. (New York, NY, USA) and DiOC18(7) from ThermoFisher Scientific (Waltham, MA, USA). All three-cell lines used colon cancer (CT26WT), and osteosarcoma (K7M2-WT) and a pre-osteoblast cell line (MC3T3-E1) were purchased from ATCC (Manassas, VA, USA).

### 2.2. Production of Shortened HNTs

**Figure 1:**
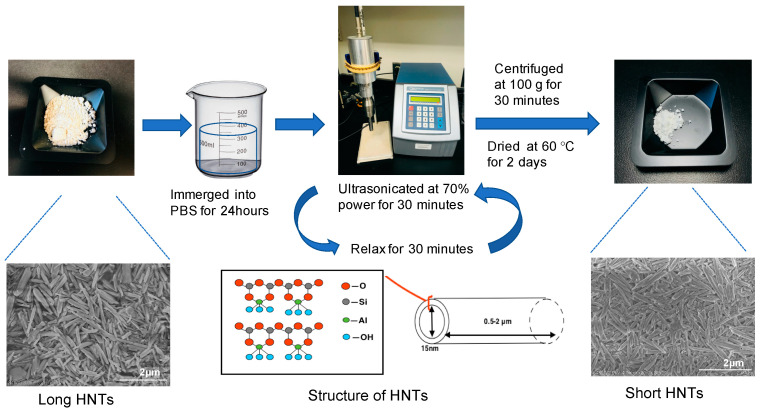
The process of reducing the length of halloysite nanotubes (HNTs). Between each step, the composites were washed and filtered using methanol, sodium chloride, a sodium bicarbonate solution, and distilled water (DI).

Commercial-grade HNTs (10 g, Sigma Aldrich, St. Louis, MO, USA) were immersed in 50 mL PBS for 24 h and ultra-sonicated for 30 min using a sonicator (Qsonica, Newtown, CT, USA) at 70% power. The solution was allowed to set for 30 min, HNTs were then ultra-sonicated for another 30 min. Then, the mixture was transferred to 50 mL centrifuge tube and centrifuged at 100× *g* for 10 min. The pelleted deposits were collected and dried at 60 °C for 2 days. See Figure 1 for details regarding this procedure.

### 2.3. bi-HNTs Synthesis

**Figure 2:**
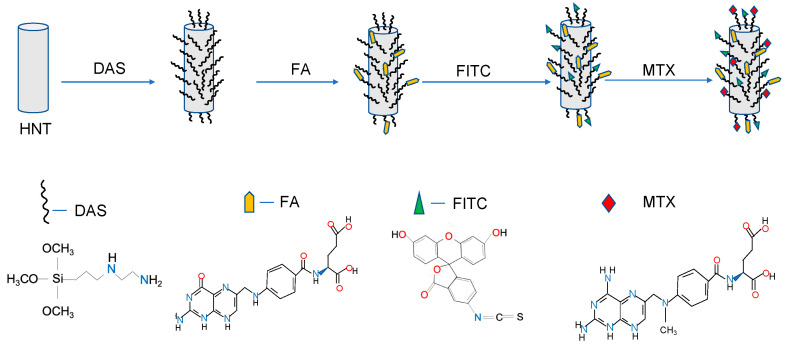
Schematic representation of the conjugation of both FA and FITC to DAS which is attached to the HNT surface. Methotrexate (MTX) was vacuum-doped into the HNT lumen. DAS = *N*-[3-(trimethoxysilyl)propyl) ethylenediamine, FA = folic acidity, FITC = fluorescein isothiocyanate.

bi-HNTs were synthesized using the same methods as described in a previous study (Figure 2) [16]. Briefly, HNTs (Sigma Aldrich, St. Louis, MO, USA) were reacted at reflux with *N*-[3-(trimethoxysilyl) propyl] ethylenediamine (DAS) (Sigma Aldrich, St. Louis, MO., USA) in toluene for 24 h. Then, the HNT-DAS composite was reacted with FA (Sigma Aldrich, St. Louis, MO, USA) in the presence of 1-ethyl-3-(3-dimethylaminopropyl) carbodiimide (EDC) (Sigma Aldrich, St. Louis, MO, USA) in DI water overnight. Finally, the HNT-DAS-FA composite was complexed with FITC (Sigma Aldrich, St. Louis, MO, USA) in acetone (Granger, Lake Forest, IL, USA) overnight.

### 2.4. bi-HNTs Characterization

#### 2.4.1. Fourier-Transform Infrared Spectroscopy (FTIR)

The Infrared spectrum was recorded at a resolution of 4 s^−1^ with 16 scans using a Thermo Scientific NICOLET™ IR100 FT-IR Spectrometer (Thermo Fisher Scientific; Waltham, MA, USA). Thermo Scientific OMNIC™ software (Waltham, MA., USA) was used to study the stretched bands.

#### 2.4.2. Scanning Electron Microscopy (SEM)

SEM images of original size of HNTs and shortened HNTs were analyzed by ImageJ (NIH, Rockville, MD, USA) and also used to measure particle size.

#### 2.4.3. X-ray Diffraction (XRD)

XRD patterns of HNTs were obtained by Bruker D8 Discover diffractometer (San Jose, CA, USA) equipped with a copper anode X-ray source and a general area detector. The power applied for all tests was 40 kV with 40mA.

### 2.5. Cell Culture

Murine colorectal cancer CT26WT, osteosarcoma cells K7M2-WT, and pre-osteoblast cells MC3T3-E1 came cryopreserved from ATCC. Cryovials were thawed and allowed to equilibrate in a water bath, all the cells were cultured in 25 cm^2^ tissue culture flask and incubated at 37 °C under humidified 5% CO_2_ and 95% air. CT26WT cells were cultured in RPMI 1640 medium, osteosarcoma cells were cultured in DMEM (Thermo Fisher Scientific, Waltham, MA, USA) and pre-osteoblast cells were cultured in alpha-MEM (Thermo Fisher Scientific, Waltham, MA, USA), all the medium containing 10% FBS and 1% penicillin. Subconfluent cells were passaged with 0.25% trypsin, collected by centrifugation, suspended in cell culture medium and cultured at a 1:4 split into 25 cm^2^ tissue culture flasks. All three types of cells through passaged four times before use.

### 2.6. MTS Assay

Cells were seeded into 48-well plate at a concentration of 1 × 10^5^ cells/well and cultured for 24 h. Then cells were incubated in cell media that contained different concentrations of pure HNTs or functionalized HNTs (0, 50, 100, 150, 200, 250 μg/mL), respectfully. MTS stock solution (Thermo Fisher Scientific, Waltham, MA, USA) (40 μL) were added to each well and cultured for 2 h at 37 °C in darkness. 200 μL of supernatant of each sample were transferred to 96-well plates and read absorbance values at 490 nm by microplate reader (Thermo Fisher Scientific, Waltham, MA, USA).

### 2.7. XTT Assay

Cells were seeded into 48-well plate at a concentration of 1 × 10^5^ cells/well and cultured for 24 h. 100 μL of cell suspension were added into 96-well tissue culture plates, then added with 25 μL activated XTT solution (XTT Cell Viability Assay Kit, Biotium, Fremont, CA, USA) and incubated for 2 h. Absorbance values were measured at 450 nm; background absorbance was measured at 630 nm. The final normalized absorbance values were obtained by subtracting background absorbance from signal absorbance.

### 2.8. Cell Uptake Efficiency 

CT26WT cells were seeded into 48 wells at the concentration of 1.5 × 10^5^ cells/well and incubated for 24 h. Then, bi-HNTs were added into cell culture medium at finial concentration of 25 μg/mL and incubated for another 24 h. After three times wash with fresh RPMI 1640, cell fluorescent intensities were measured by fluorescent microplate reader at 490/525 nm (Ex/Em). The cell numbers were measured.

### 2.9. Cell Uptake Mechanism

CT26WT cells were seeded into 24 wells at the concentration of 2 × 10^5^ cells/well and cultured for 24 h. Two inhibitors, chlorpromazine and filipin, were selected to evaluate the bi-HNTs cell uptake mechanism. Chlorpromazine selectively inhibits clathrin-dependent endocytosis and filipin inhibits caveolae-dependent endocytosis. Cells were pre-cultured with CPZ (Sigma Aldrich, St. Louis, MO, USA) (7 μg/mL) or filipin (5 μg/mL) for 2 h. Cells treated without any inhibitor set as the control. bi-HNTs (25 μg/mL) were added to each well and incubated for another 24 h. Fluorescent intensities expressed by cells were measured as above.

### 2.10. Multi-Photon Imaging

CT26WT cells were seeded on a glass slide at a concentration of 1 × 10^5^ cells/mL and cultured for 24 h. bi-HNTs were added to the cell culture plate (25 μg/mL) and continually incubated for 12 h. DiOC18(7) (DiR) (ThermoFisher Scientific) working solution was prepared by following the company provided protocol. Cells were washed with DPBS for 3 times, then the DiR working solution was added into cell culture well (1 μL/mL) and incubated for 2 h. Cells were then fixed by 4% paraformaldehyde (Sigma Aldrich, St. Louis, MO, USA) for imaging by an in-house assembled multi-photon microscopy. Three-dimensional images were acquired using a Vivo-2 upright, multiphoton microscope system (Intelligent Imaging Innovations, Inc. (3i), Denver, Colorado, USA) with GaAsP photomultipliers (Hamamatsu, Tokyo, Japan), a Pockels cell (Conoptics, Inc., Danbury, CT, USA) to control laser power, and an ELWD 40×/0.6 NA objective lens (Nikon Instruments, Inc., Tokyo, Japan). Excitation of fluorescence was provided by a Chameleon Vision-2 multiphoton laser (80 MHz, Coherent. Inc., Santa Clara, CA, USA) tuned to 850 nm. Slidebook 6 software (3i) (Conoptics, Inc., Danbury, CT, USA) was used to control the microscope and multiphoton laser systems and to acquire images. Images were scanned in three dimensions with x- and y-dimensions of 248 μm (1024 × 1024-pixel resolution), with z-steps of 2 μm (z stack) to produce z stacks of 36 and 46 μm to ensure that all cells were captured within the volume. Pixel dwell time was set to 2 μL with pixel averaging of 5/scan to acquire z stack images [17,18].

### 2.11. Apoptosis and Necrosis

CT26WT cells were seeded into 24 wells at a concentration of 2 × 10^5^ cells/well and cultured for 24 h. Then, a high dosage of bi-HNTs were added into the cell culture medium (150 μg/mL) and incubated for 24 h. Then 3 × 10^5^ cells were collected and suspended in 500 µL of 1× binding Buffer, then mixed with 5 µL of Annexin V-FITC and 5 µL of propidium iodide (Sigma Aldrich, St. Louis, MO, USA). Cell suspension mixtures were incubated in the dark at room temperature for 5 min. Then samples were examined by flow cytometry (Thermo Fisher Scientific, Waltham, MA, USA) at 488/530 nm (Ex/Em). Beside the resuspended cells, we kept a portion of attached cells in 24 wells and stained by 5 µL of Annexin V-FITC and 5 µL of propidium iodide as well. 

### 2.12. Drug Loading

Methotrexate (MTX) (Sigma Aldrich, St. Louis, MO, USA) was dissolved in PBS (1 mg/10 mL) and mixed with bi-HNTs (200 mg) and keep stirring for 12 h, then, the mixture was vacuumed for 24 h. After centrifugation, the supernatant liquid was collected and stored at −20 °C for drug loading efficiency determination, and the bottom deposited bi-HNTs were washed by PBS for 3 times and air-dried, the final product was collected for further study.

### 2.13. Drug Loading Efficiency

The supernatant was diluted with PBS at ratio of 1 (supernatant): 20 (PBS). Then the drug concentration in supernatant was determined by MTX Elisa kit (ENZ-KIT 142-0001, Enzo Life Science, Farmingdale, NY, USA). Experiment procedure followed the manufactory product manual. First, MTX standard (S1-S6) was prepared by diluting the company supplied lyophilized MTX to suggested concentrations (1000 ng/mL, 166.7 ng/mL, 27.8 ng/mL, 4.6 ng/mL, 0.77 ng/mL, 0.13 ng/mL). Then, 100 μL of standard solution and samples were added into 96 wells, 100 μL of assay buffer 13 was added to one well and set as S0 (0 ng/mL standard), and 150 μL of assay buffer 13 was added to another well set as NSB well. 50 μL of MTX antibody was added to all wells except for the NSB and blank, and this 96 well plat was sealed and incubated at room temperature on a plate shaker (Thermo Fisher Scientific, Waltham, MA, USA) for 30 min at ~500 rpm.

Then, all the wells were washed by wash buffer for 3 times. Followed with addition of 100 μL of MTX conjugated solution to all the wells except the blank, this plate was incubated at room temperature as before. After another 3 times wash, 100 μL of substrate solution was added to all wells and went through the final incubation as same as above. Finally, add 100 μL of stop solution to all wells and read the plate at 450 nm. According to the standard curve to calculate the MTX concentration of supernatant.

The drug loading efficiency was determined by following equation:

Loading Efficiency = (Total amount of MTX − supernatant MTX)/Total amount of MTX × 100%(1)

### 2.14. Drug Release Profile

MTX-loaded bi-HNTs (20 mg) were mixed with 2 mL PBS in a tube and incubated at room temperature on a rocking shaker. After incubated for different time periods (0.5, 1, 2, 4, 6, 8, 14, 24, 32, 48, 56, 64, 72, 80, 88, and 96 h) tube was centrifuged and 1 mL of supernatant was collected, and 1 mL fresh PBS refilled to tube. The collected samples were stored at −20 °C until all the time periods samples were collected and measured by MTX Elisa kit as above.

### 2.15. MTX-bi-HNTs Targeting

Methotrexate loaded bi-HNTs (50 μg/mL) were co-cultured with CT26WT, K7M2-WT, and MC3T3-E1 pre-osteoblasts separately in 48 wells tissue culture plates (1 × 10^5^ cells/well). After 24 h incubation, cell viability of each cell type was assessed by MTS agent as above. Cells cultured without HNTs set as control.

### 2.16. Statistical Analysis

Data were obtained from three parallel experiments and are expressed as mean ± standard deviation (S.D.). Each experiment was repeated for three times to check the reproducibility if not otherwise stated. Statistical analysis was performed by two-tailed Student’s *t*-tests between two groups. The significant level was set as *p* < 0.05.

## 3. Results

### 3.1. Optimal Dosage Determination

**Figure 3:**
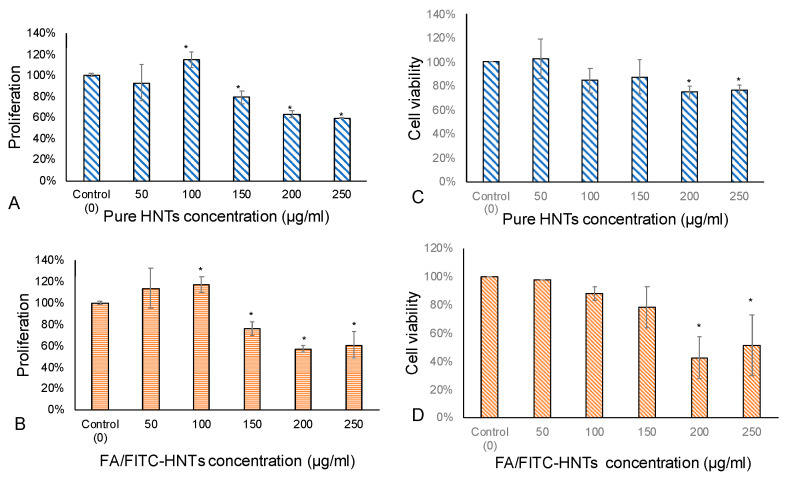
XTT Cell Proliferation Assay. CT26WT cells were cultured with pure HNTs (**A**) and bi-HNTs (**B**) at different concentrations. Cell proliferation at each different concentration was calculated with comparison to the control group. CT26WT cells cultured with pure HNTs (**C**) and bi-HNTs (**D**) at different concentrations. Cell viability at each different concentration point was calculated by comparison to the control group. (error bar with standard deviation, *n* = 6, * represents *p* < 0.05).

In our previous study [16], we assessed bi-HNTs cytotoxicity and observed that cytotoxicity increased with bi-HNT concentration. In order to confirm the optimal dosage range for bi-HNTs, we used an MTS assay and XTT assay to assess cell proliferation and viability with cells co-cultured with pure HNTs and bi-HNTs at different concentrations. Both tests presented similar results: cell viability changed with the concentration of HNTs nanoparticles. In the MTS assay (Figure 3A,B), cell proliferation was improved when the concentration of nanoparticles was below 100 mg/mL. However, when the concentration was increased to 150 mg/mL, the proliferation ability decreased by 20%. XTT assay showed similar results (Figure 3C,D), cell viability decreased with the increasing concentration of HNTs. However, the XTT assay showed that CT26WT cells have some tolerance to higher concentrations of HNTs, with significant cytotoxicity exhibited above 200 mg/mL. The cumulative results suggest that the highest dosage of bi-HNTs is 150 mg/mL.

### 3.2. The Effect of Nanoparticle Size on the Interaction between bi-HNTs and Cells

#### 3.2.1. Nanoparticle Size Determination

Halloysite nanotubes are naturally formed nanoparticles with an original length between 1–1.5 μm. Recently, several studies have used shortened HNTs [11,19,20]. Rong et al. introduced a detailed procedure to produce HNTs that are uniform in length [20]. We fabricated shortened HNTs by ultra-sonication (Figure 1). Dynamic light scattering (DLS) is the most commonly used.

**Figure 4:**
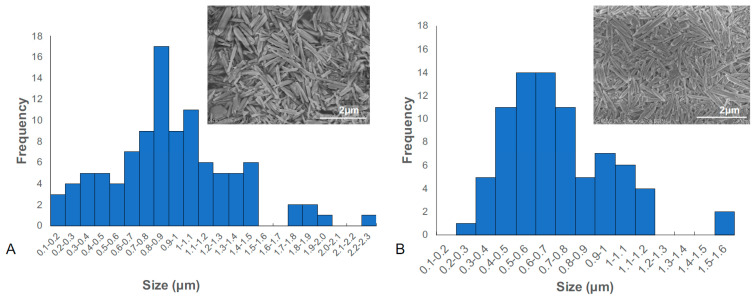
Histogram graph of size distribution of long HNTs (**A**) and shortened HNTs (**B**).

Technique to determine nanoparticle size, however, DLS analysis requires spherical nanoparticles instead of virgate nanotubes. Thus, we used SEM micrographs of our HNT samples NIH Image J to measure their size and plotted the data into histogram graph (Figure 4). As it showed in Figure 4, the length of commercial purchased HNTs distributes in a very wide range with the average size of 0.914 ± 0.45 μm, *n* = 102 (Figure 4A), while the size of shorten HNTs was 0.715 ± 0.25 μm, *n* = 80 (Figure 4B). Comparing the two different sources of HNTs, we observed the size distribution of shortened HNTs to be more restricted in variation, which means they are more uniform in overall size. HNTs with a uniform size will provide a more accurate means for evaluating its effect on cellular behavior.

#### 3.2.2. Cellular Interaction after Exposure to Long and Shortened HNTs

**Figure 5:**
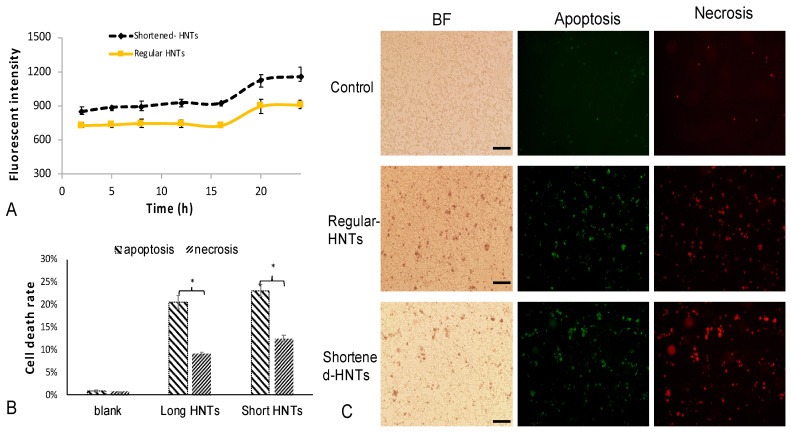
(**A**). Fluorescent intensity of FITC detected from CT26WT cells after 24 h incubation time (cell number for each test = 4.46 × 10^5^ ± 0.063 × 10^5^, each group had 9 tests, error bar with standard deviation). (**B**). The summary of flow cytometry results for apoptosis and necrosis analysis. The detailed flow cytometry results showed in Supplementary Information Figure S1. (cell number for each test = 3 × 10^5^, each group had 6 tests, error bar with standard deviation, * represents *p* < 0.05) (**C**). Fluorescent pictures for apoptosis & necrosis analysis, the green color represents apoptosis and the red color represents necrosis. The first column represents brightfield images (scale bar with 100 μm).

We hypothesized that different sized HNTs would affect cellular behavior, including our bi-functionalized HNTs. Cell up taking efficiency for two different sized HNTs was analyzed. We monitored the fluorescent intensity changes of cells (cell number = 4.46 × 105 ± 0.063 × 105) in 24 h (Figure 5A). Cellular uptake efficiency for both sizes of HNTs exhibited similar changes along with time. In the end, higher amounts of shortened HNTs are detected in cells, indicating that the smaller size of HNTs is more easily absorbed by cells.

#### 3.2.3. Apoptosis and Necrosis

Previous cell cytotoxicity studies showed that cell viability decreased significantly when the concentration of HNTs reached 150 mg/mL [16]. To distinguish the mechanism of cell death, we examined cell death due to apoptosis or necrosis. CT26WT cells were incubated with long and short bi-HNTs; cell death was assessed using the Annexin V-FITC Apoptosis Kit. Apoptosis and necrosis were assessed by flow cytometry. The detail res as shown in Figure 5B, 20% ± 1.5% of cell death was caused by apoptosis and 10% ± 0.5% was due to necrosis when cells were cultured with long bi-HNTs. Cells cultured with shorted bi-HNTs showed apoptotic cell death at 23% ± 1.3% and 13% ± 0.6% due to necrosis. Thus, cell death was primarily due to apoptosis for long and shortened bi-HNTs. As seen in Figure 5, cells accumulated intracellularly shorter bi-HNTs as compared to long bi-HNTs and shortened bi-HNTs resulted in an overall higher cell death suggesting excessive cellular accumulation of bi-HNTs leads to apoptosis.

### 3.3. Intracellular Location of bi-HNTs

**Figure 6:**
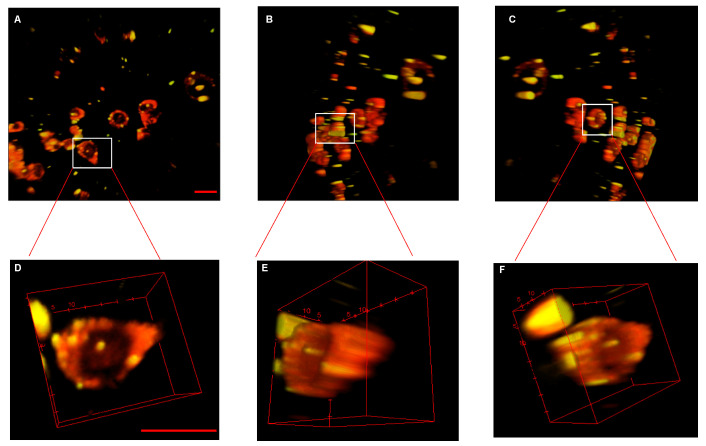
Multi-photon pictures of cells after exposure to bi-HNTs, pictures were analyzed with NIH Image J. Cell membranes were stained by DiR dye and exhibited a red color at wavelength of 850 nm. At this wavelength FITC exhibited a yellow color. The 3D pictures were captured at 36 microns in z with a 2 micron separation in each z step. (**A**) is the front view of the 3D picture for multiple cells, (**B**,**C**) are side views of the 3D picture. (**D**–**F**) are the zoom in pictures of the marked cell. (Scale bar with 50 μm).

In order to confirm the intracellular location of bi-HNTs, we co-cultured bi-HNTs with CT26WT cells for 24 h and cells were then incubated with DiR for another 2 h. Multi-photon microscopy imaging showed cell were stained red with each reddish circular structure representing the plasma membrane; the yellow particles represent bi-HNTs (Figure 6). Multi-photon images of labeled cells showed that many bi-HNTs were present within the plasma membrane and some had accumulated in the cytoplasm. The results further confirmed the targeting capacity of bi-HNTs in vitro.

### 3.4. Targeted Drug Delivery

The experiments described above support the observation that our bi-HNTs were successfully taken up by colon carcinoma cells (CT26WT). As the bi-HNTs were designed as a drug delivery system, we selected methotrexate (MTX) as model drug and loaded into bi-HNTs. Methotrexate is a commonly used anti-cancer drug to osteosarcoma. However, it is well-known for severe side effects. A cancer target drug delivery system is a promising strategy to reduce side effects and improve drug efficiency.

#### 3.4.1. Folic Acid Coating and Drug Releasing

**Figure 7:**
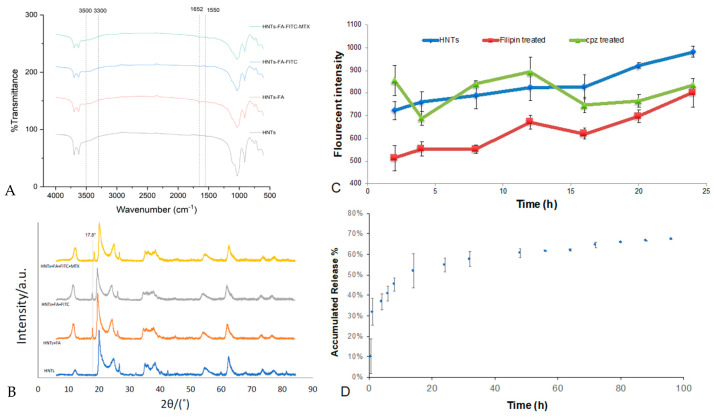
(**A**) FTIR detection of pure HNTs, HNTs-FA, FA/FITC-HNTs and FA/FTIC/MTX-HNTs. (**B**) XRD patterns of pure HNTs, HNTs-FA, FA/FITC-HNTs and FA/FTIC/MTX-HNTs. (**C**) Fluorescent intensity of FITC included in cells that pretreated by chlorpromazine (CPZ) or filipin and co-cultured with bi-HNTs for different time periods. (error bar with standard deviation, *n* = 6) (**D**) Accumulated drug release profile of methotrexate in 96 h. (Error bar with standard deviation, *n* = 3).

The chemical modification of bi-HNTs were detected by FTIR. Halloysite is an aluminosilicate clay mineral (Al_2_Si_2_O_5_(OH)_4_), the Si-O stretching vibrations and Al–OH vibrations were represented at 1000–1130 cm^−1^. Folic acid, FITC and MTX containing –NH and –OH bonds, their characteristic absorption is in the range of 3300–3500 cm^−1^, NH_2_ scissoring at 1550 cm^−1^ and NH associated bending at 1652 cm^−1^. The deformation in 3300–3500 cm^−1^ and 1652 cm^−1^ indicated the successful grafting of FA, FITC, and MTX (Figure 7A) [21]. The XRD patterns also present similar results. In Figure 7B, except for the HNTs profile there is a sharp peak appeared at 2θ = 17.8° for other groups, which is the diffraction peaks of folic acid. The slight shift of this peak at HNTs + FA + FITC + MTX profile may due to the methotrexate loading. The loading efficiency of MTX in bi-HNTs was 34.74% ± 3.5%. Its drug release profile is presented in Figure 7D. Even through, there was a burst release during the first 20 h, this drug delivery system provided sustained drug release for more than 96 h.

#### 3.4.2. Cellular Uptake Mechanisms

Folic acid coated on bi-HNTs was designed to bind to folate receptors expressed by cancer cells. In the binding process, folic acid initiates cellular uptake mechanisms in two different ways: clathrin-mediated endocytosis or caveolae-mediated endocytosis. Chlorpromazine (CPZ) has the ability to disrupt clathrin-mediated endocytosis; while filipin inhibits caveolae formation. CT26WT cells were pretreated with these inhibitors and co-cultured in the presence of bi-HNTs. Their final fluorescent intensity was recorded, and the results are shown in Figure 7C. The addition of CPZ promoted HNT uptake in first 12 h, then the cell uptake decreased over time. On the other hand, filipin provided uptake inhibition during the entire testing time period. This indicates cellular absorption of bi-HNTs may mainly depend on caveolae-mediated endocytosis assist by clathrin-mediated endocytosis.

#### 3.4.3. In Vitro Targeted Drug Release-Cellular Specificity

**Figure 8:**
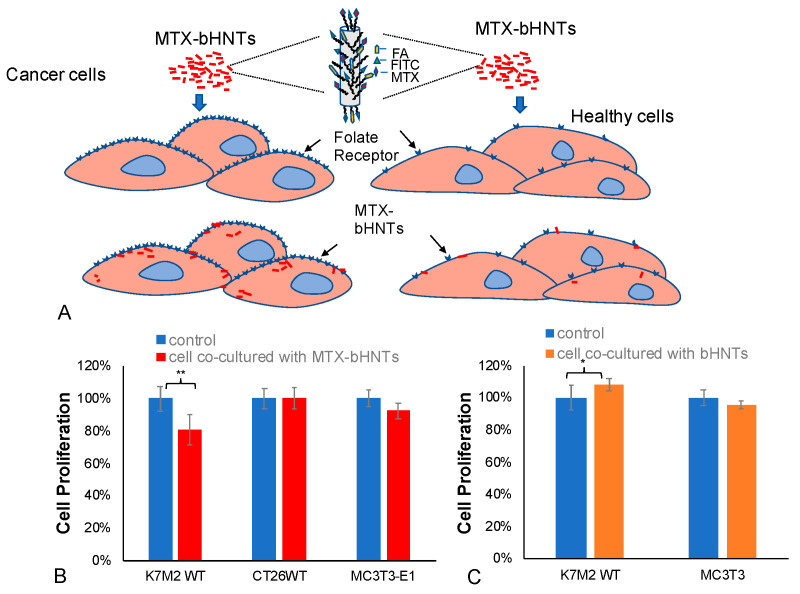
(**A**) Graphic depiction of osteosarcoma cells (K7M2WT), murine colon carcinoma cells (CT26WT) and preosteoblast cells (MC3T3) co-cultured with bi-HNTs. (**B**) Cell proliferation data after all three cell types were co-cultured with 50 μg/mL drug loaded bi-HNTs for 24 h. (**C**) Cell proliferation of above 3 types of cells after co-cultured with 50 μg/mL bi-HNTs for 24 h. (* represents *p* < 0.05, ** represents *p* < 0.005).

The bi-HNTs drug delivery system was designed to deliver drugs to specific cancer cells. Methotrexate is one of the most widely used drugs in treating osteosarcoma. Like many cancer drugs, it does have adverse side effects. In this experiment, the specific targeting of cancer cells versus normal cells was addressed. A pre-osteoblast cell line (MC3T3-E1), and two cancer cell lines, a colon cancer cell (CT26WT) and an osteosarcoma cell line (K7M2-WT), were selected to assess the targeting potential of bi-HNTs, and drug effectiveness in inhibiting cellular proliferation, the main effect of MTX (Figure 8A). The results suggest that MTX-loaded bi-HNTs (MTX-bi-HNTs) significantly inhibited osteosarcoma cell proliferation, and, at a low concentration (50 μg/mL). The other two cell types were not significantly affected by exposure to MTX (Figure 8B).

In order to clarify that inhibition of cell growth was caused by MTX instead of bi-HNTs, we analyzed cell viability by co-culturing cells with bi-HNTs but without MTX. The results showed that none of the osteosarcoma (K7M2-WT) or pre-osteoblast cells (MC3T3-E1) were affected by bi-HNTs (Figure 8C). This finding is consistent with our previous study using CT26WT; a low dose of bi-HNTs did not elicit any cytotoxicity (Figure 3). Collectively, these results demonstrate the effectiveness of MTX loaded bi-HNTs in selectively targeting osteosarcoma cells in delivery of MTX, inhibiting cell proliferation.

## 4. Discussion

Many studies have exploited halloysite as a nanocontainer loading chemotherapeutic agents into the HNT lumen [19,20,21,22] and adding doped HNTs to a range of polymers for anti-cancer drug delivery [23,24,25,26]. Other studies have focused on using surface modification of HNTs for use as a nanocarrier for anti-cancer drug delivery [20,27,28,29]. A commonly used strategy is to functionalize HNTs with an –NH2 group by using aminopropyltriethoxysilane (APTES). In a study by Guo et al. (2012), FA and magnetite nanoparticles (Fe_3_O_4_) were successfully grafted onto the HNT surface. The coated Fe_3_O_4_@HNTs exhibited a pH-sensitive drug release under the electrostatic interaction between the cationic and HNTs [20]. Coating nanotubes with a polymer shell is another mechanism, as shown by Li et al. (2018). They examined the potential of chitosan grafted onto HNTs as a nano-formulation for the anti-cancer drug curcumin [27].

Fewer studies have attempted to use modified HNTs as a mechanism for the intracellular delivery of anti-cancer agents [16,28,29,30] Dzamukova et al. (2015) used physically adsorbed dextrin end stoppers to secure the intracellular release of brilliant green [28]. Tagged halloysite nanotubes were also used as carriers for intercellular delivery to brain microvascular endothelium [29]. Kamalieva et al. (2018) studied the intracellular pathway of HNTs for potential application for antitumor drug delivery using human adenocarcinoma epithelial cells (A549) [30].

MTX is an FDA approved anti-cancer drug commonly used in the treatment of osteosarcoma. In a recent study, MTX-doped HNTs were coated with polyelectrolytes (PE), polyvinylpyrrolidone, and polyacrylic acid, and methotrexate [31] was infused within the coated layers. MTX release and cytotoxicity studies showed effectiveness in inhibiting osteosarcoma cell growth, and inhibition continued after PE/MTX-coated halloysite nanotubes were added to a polymer, Nylon-6.

Due to high chemical similarity in the structure between MTX and FA, several studies have shown that MTX modified nanoparticles have specificity for tumor cells [32,33,34]. Folic acid has a high binding affinity for the folate receptor, which is overexpressed in numerous cancers, including ovarian, endometrial, and renal carcinoma, lung, breast, and brain cancers [35]. MTX-loaded PEGylated chitosan nanoparticles had a higher cellular uptake efficiency compared to the FA-tagged group [36]. The high receptor affinity and overexpression enables folate-based nanoparticles to be highly specific, targeting tumor sites, and have great potential as a therapeutic application. FA-conjugated chitosan oligosaccharide-magnetic HNTs were studied as a delivery system for camptothecin, an anti-cancer drug [37]. Wu et al. (2018) also functionalized the HNT surface with APTES for conjugation of PEG and folic acid and subsequently loaded HNTs with doxorubicin [38]. The limitation of their study was the reported low drug loading efficiency, which was only 3%. In contrast, in this study, the loading efficiency of methotrexate was over 30%. In a recent report, HNTs had a loading efficiency of indocyanine green (ICG) as high as above 60% [39]. The variation in drug loading capability may be related to the different modification strategies used and the molecular size of drugs loaded.

The intracellular uptake pathway of HNTs was previously studied by Liu et al. [40]. HNTs were modified with APTES and labeled with FITC. Cells were treated with four different inhibitors, respectively, and then co-cultured with the FITC functionalized HNTs. They found both clathrin- and caveolae- dependent endocytosis were involved in the internalization of HNTs [40]. Liu’s group also found that microtubules and actin microfilaments transported HNTs with the involvement of the Golgi apparatus and lysosome. Our results are consistent with their study. However, we observed that caveolae-mediated endocytosis was the primary mechanism of cellular import, and clathrin-mediated endocytosis was a secondary mechanism. Different surface modification strategies and cell types may be the possible explanation for the different results obtained in these studies and those reported here.

Even after pretreatment with chlorpromazine (CPZ), cytoplasmic accumulation of bi-HNTs was much more significant than the control group after the initial 12 h incubation time. One possible explanation is that disruption of clathrin-mediated endocytosis caused by CPZ induced caveolae to expand or more endocytic vesicles formed to internalize bi-HNTs. However, the overloading capability of caveolae reached capacity after several hours. Cells were unable to process the bi-HNTs loaded vesicles, so some were released from the cells. There is no report on the interaction between clathrin-and caveolae-mediated endocytosis during HNT uptake. Therefore, the detailed mechanism behind this phenomenon remains to be determined.

Our data further confirmed that bi-HNTs served well as a drug carrier and provided sustained MTX release time and showed selective binding to osteosarcoma cells. In contrast, non- osteosarcoma cells exposed to MTX were unaffected. We further showed that HNT size was also played a critical role in cellular uptake. Cellular uptake of smaller sized bi-HNTs was observed in greater amounts than unmodified HNTs. Increased intracellular accumulation may contribute to cell death through disruption of normal cellular metabolism resulting in cell death. Therefore, the application dosage of bi-HNTs should decrease with the size diminution; in other words, the smaller size of bi-HNTs has a higher working efficiency.

Even though the drug-loaded bi-HNTs has targeted FA receptors and successfully inhibited cell proliferation, this study lacks the comparison between MTX and MTX-loaded bi-HNTs. As an FDA approved anti-cancer drug, MTX would inhibit cell proliferation for sure. As a drug delivery system, bi-HNTs could extend the drug release time. We hypothesis MTX-loaded bi-HNTs would limit cell proliferation for a longer time compared to pure MTX treatment.

## 5. Conclusions

bi-HNTs were cytocompatible in an appropriate dosage range, permitting a decrease in MTX dose when used with size reduced bi-HNTs. The results further showed that caveolae-mediated endocytosis is the main uptake pathway of bi-HNTs, and multi-photon images confirmed cellular uptake of bi-HNTs. This modified nanocarrier also showed excellent MTX drug loading capability. Most importantly, drug-loaded MTX-bi-HNTs exhibited an excellent selectively targeting ability in vitro, as both colon carcinoma and osteosarcomas cells demonstrated bi-HNT uptake. Due to the drug specificity, the inhibition of cell proliferation was restricted to osteosarcomas, while murine colon carcinoma cells and pre-osteoblasts were not affected. Surface modification of HNTs with two ligands—FA and FITC—provided a selective targeting ability for osteosarcoma cells and an imaging vehicle for tracking bi-HNTs, and may have potential as an alternative treatment for osteosarcoma.

## Supplementary Materials

The following are available online at https://www.mdpi.com/1999-4923/12/10/962/s1. Figure S1: A. Flow cytometer observation of each treatment. B. The selected dot were continued applied with FITC filter for apoptosis (first row) and PE filter for necrosis (second row).
